# Gastrocnemius Medialis Muscle Geometry and Extensibility in Typically Developing Children and Children With Spastic Paresis Aged 6–13 Years

**DOI:** 10.3389/fphys.2020.528522

**Published:** 2020-11-23

**Authors:** Guido Weide, Peter A. Huijing, Lynn Bar-On, Lizeth Sloot, Annemieke I. Buizer, Jules G. Becher, Jaap Harlaar, Richard T. Jaspers

**Affiliations:** ^1^Laboratory for Myology, Department of Human Movement Sciences, Faculty of Behavioral and Movement Sciences, Amsterdam Movement Sciences, Vrije Universiteit Amsterdam, Amsterdam, Netherlands; ^2^Department of Rehabilitation Medicine, Amsterdam UMC, Vrije Universiteit Amsterdam, Amsterdam Movement Sciences, Amsterdam, Netherlands; ^3^Department of Rehabilitation Sciences, Katholieke Universiteit Leuven, Leuven, Belgium; ^4^Emma Children’s Hospital, Amsterdam UMC, University of Amsterdam, Amsterdam, Netherlands; ^5^Department of Biomechanical Engineering, Delft University of Technology, Delft, Netherlands

**Keywords:** cerebral palsy, muscle morphology, muscle architecture, spasticity, ultrasound

## Abstract

Gait of children with spastic paresis (SP) is frequently characterized by a reduced ankle range of motion, presumably due to reduced extensibility of the triceps surae (TS) muscle. Little is known about how morphological muscle characteristics in SP children are affected. The aim of this study was to compare gastrocnemius medialis (GM) muscle geometry and extensibility in children with SP with those of typically developing (TD) children and assess how GM morphology is related to its extensibility. Thirteen children with SP, of which 10 with a diagnosis of spastic cerebral palsy and three with SP of unknown etiology (mean age 9.7 ± 2.1 years; GMFCS: I–III), and 14 TD children (mean age 9.3 ± 1.7 years) took part in this study. GM geometry was assessed using 3D ultrasound imaging at 0 and 4 Nm externally imposed dorsal flexion ankle moments. GM extensibility was defined as its absolute length change between the externally applied 0 and 4 Nm moments. Anthropometric variables and GM extensibility did not differ between the SP and TD groups. While in both groups, GM muscle volume correlated with body mass, the slope of the regression line in TD was substantially higher than that in SP (TD = 3.3 ml/kg; SP = 1.3 ml/kg, *p* < 0.01). In TD, GM fascicle length increased with age, lower leg length and body mass, whereas in SP children, fascicle length did not correlate with any of these variables. However, the increase in GM physiological cross-sectional area as a function of body mass did not differ between SP and TD children. Increases in lengths of tendinous structures in children with SP exceeded those observed in TD children (TD = 0.85 cm/cm; SP = 1.16 cm/cm, *p* < 0.01) and even exceeded lower-leg length increases. In addition, only for children with SP, body mass (*r* = −0.61), height (*r* = −0.66), muscle volume (*r* = − 0.66), physiological cross-sectional area (*r* = − 0.59), and tendon length (*r* = −0.68) showed a negative association with GM extensibility. Such negative associations were not found for TD children. In conclusion, physiological cross-sectional area and length of the tendinous structures are positively associated with age and negatively associated with extensibility in children with SP.

## Introduction

Childhood spastic paresis (SP) is characterized by an upper motor neuron impairment, which is associated with gait abnormalities and limitations in mobility. Most children with SP are diagnosed with spastic cerebral palsy, which is an umbrella term for a clinically heterogeneous syndrome caused by congenital brain malformation or an acquired dysfunction of the immature brain during pregnancy, around birth, or during early development (Rosenbaum et al., 2007). Brain lesions in cerebral palsy are visible on magnetic resonance imaging (MRI). However, some children show similar clinical features of SP without brain abnormalities. Several genetic abnormalities have been identified as a cause for Hereditary Spastic Paresis (HSP) (Parodi et al., 2018). However, presently not all genetic or metabolic defects have been discovered. We label a non-progressive upper motor neuron syndrome, without MRI abnormalities or a known genetic cause, a SP of unknown etiology.

Children with spastic involvement of the lower leg often develop equinus gait, which is characterized by ankle plantar flexion in the mid-stance phase and limited push-off power in walking (Voorman et al., 2007; Gage, 2009; Ballaz et al., 2010; Dallmeijer et al., 2011). Without treatment, walking ability and ankle range of motion (ROM) impairments worsen with age (Beckung et al., 2007; Nordmark et al., 2009). Clinical interventions often involve a combination of physiotherapy, orthoses, serial casting, spasticity treatment like Botulinum Toxin-A (BoNT-A) intramuscular injections (intrathecal), baclofen administration, selective dorsal rhizotomy and orthopedic surgery (Koman et al., 2004). Although the above interventions improve passive ankle ROM in the short-term (Nieuwenhuys et al., 2016), recurrence in the long-term has frequently been reported (Fry et al., 2007; Spijker et al., 2009; Tedroff et al., 2009).

Increased resistance to ankle dorsal flexion in passive stretch (ankle joint hyper resistance) is an often encountered problem in children with spastic cerebral palsy. This problem has also been reported in children with HSP (Marsden et al., 2012). Ankle joint hyper-resistance to dorsal flexion is predominantly reported to be related to increased triceps surae (TS) hyper-resistance (van den Noort et al., 2017). This muscular hyper-resistance primarily relates to disturbances in muscle excitation in SP, such as hyperreflexia and involuntary background excitation (van den Noort et al., 2017). Secondary to these disturbances related to muscle excitation, muscle hyper-resistance is influenced by changes in morphological characteristics. In animal experiments, muscle geometric characteristics have been shown to be major determinants of active and passive length-force characteristics (Woittiez et al., 1983). Geometrical characteristics of the gastrocnemius medialis muscle (GM) have been investigated extensively in animal and human studies (Huijing, 1985; Zuurbier and Huijing, 1993; Mathewson and Lieber, 2015). Studies in children with SP have shown that GM volume normalized for body mass is on average around 22% smaller than in TD children (Barber et al., 2011a, 2016). Such reduced GM volumes may be related to relatively shorter fascicles (Mohagheghi et al., 2008; Barber et al., 2011b), and/or smaller physiological cross-sectional areas (A_*fasc*_) (Lieber et al., 2003; Malaiya et al., 2007; Barber et al., 2011b). In turn, a lower optimum fascicle length, due to fewer serial sarcomeres and a smaller physiological cross-sectional area due to fewer myofibrils arranged parallel will probably affect active and passive ranges of force exertion of the muscle. In particular, lower optimum fascicle lengths will reduce both the length range of active force exertion and extensibility. A smaller A_*fasc*_ reduces optimum force and theoretically improves muscle extensibility because of a reduction of parallel arranged tissue.

To date, most studies examining the anatomy of the GM have focused on the muscle belly (see for review: Barrett and Lichtwark, 2010). The tendinous structures, and their relation to muscle belly characteristics, have not been studied extensively. Therefore, it is not well understood how altered GM geometry in children with SP affects ankle dorsal flexion hyper-resistance. Most comparisons of morphological determinants of limited ankle-hyper resistance in children with SP have been based on group differences, concealing age-related individual variations within groups. Thus, more insight in the development of GM geometry in typically developing (TD) children and in children with SP is needed.

In TD children, GM growth is related to uniform increases in physiological cross-sectional area, fascicle and tendon length (Bénard et al., 2011). To understand the underlying mechanisms of ankle joint hyper-resistance in children with SP, estimates of such GM morphological variables in children with SP, and their relation to age, body dimensions and GM extensibility are required.

The aim of this study was to compare GM geometry and GM extensibility between children with SP and TD children. In addition, we aimed to evaluate if and how alterations in GM geometry relate to GM extensibility.

## Materials and Methods

### Participants

Thirteen children between the age of 6 and 13 years with uni- or bilateral SP (GMFCS I-III), due to spastic cerebral palsy or SP of unknown etiology (no abnormalities on brain MRI, and no abnormalities on genetic testing) were selected from the patient population at the outpatient clinic of the department of rehabilitation medicine at the Amsterdam UMC (location VUmc), Amsterdam, Netherlands. Children were excluded if they had undergone any neurosurgery or orthopedic surgery in the past, or chemical denervation of the lower limb less than six months prior to the measurements. Children had to be physically capable to participate in the measurements. A convenience sample of 14 typically developing children (TD) in the same age range as the children with SP participated as controls. For both groups, we excluded children if they had any other disorders affecting the musculoskeletal system. Before taking part, written informed consent from both parents and verbal consent from participants was received. The study was approved by the medical ethics committee of the Amsterdam University Medical Centers, location VUmc. For all subjects, measurements were performed by the same assessor (GW). In the SP group, measurements were performed on the leg with the most resistance against ankle dorsal flexion, as assessed during a clinical physical examination. In the TD group, we selected the right leg. Individual participant characteristics and patient diagnoses are shown in Table 1.

**TABLE 1:** Individual participant characteristics; age, anthropometrics, patient diagnosis, and gastrocnemius medialis muscle geometry (at 0 Nm).

	Variables																		
	
Subject	Age (years)	Body mass (Kg)	Body height (cm)	Lower leg length (cm)	Body mass index (-)	Sex (m/f)	GMFCS	Etiology (CP/SP-ue)	Distribution (Uni-/Bilateral)	BoNT-A history (0, 1, > 1)	V_*GM*_ (ml)^§^	A_*fasc*_ (cm^2^)	ℓ_*fasc*_ (cm)	α_*fasc*_ (deg)	ℓ_*m*+*t*_ (cm)	ℓ_*a*+*t*_ (cm)	ℓ_*m*_ (cm)	ℓ_*t*_ (cm)	ℓ_*a*_ (cm)
CP01	6.0	21.0	120.0	25.7	14.6	f	2	SP-ue	Bi	3	32.6	7.8	4.2	10.9	26.9	22.8	13.7	13.2	9.6
CP02	8.3	23.0	125.0	27.9	14.7	f	2	CP	Bi	1	42.0	8.7	4.8	9.6	29.0	24.3	17.4	11.6	12.7
CP03	11.1	55.7	159.3	37.9	21.9	m	2	CP	Bi	1	83.9	30.7	2.7	23.9	40.1	37.6	19.5	20.6	17.0
CP04	10.1	31.5	142.0	32.4	15.6	f	1	CP	Bi	0	53.5	13.5	4.0	14.6	34.4	30.6	21.0	13.5	17.2
CP05	11.9	40.8	154.4	38.9	17.1	m	2	SP-ue	Bi	1	63.8	16.9	3.8	15.1	41.3	37.6	22.5	18.8	18.8
CP06	9.2	28.8	135.0	31.1	15.8	f	2	SP-ue	Bi	0	58.8	12.3	4.8	14.2	31.9	27.3	19.9	11.9	15.4
CP07	11.6	41.3	146.7	31.7	19.2	m	2	CP	Bi	1	52.6	13.2	4.0	16.1	33.3	29.5	17.6	15.7	13.8
CP08	12.1	30.2	129.3	29.0	18.1	m	3	CP	Bi	5	48.0	14.8	3.2	13.4	31.2	28.1	15.2	16.0	12.0
CP09	7.5	23.6	118.2	26.2	16.9	m	2	CP	Bi	1	31.7	8.5	3.7	12.5	26.5	22.9	15.5	11.0	11.9
CP10	10.8	42.0	140.0	33.4	21.4	m	2	CP	Bi	0			5.3	13.2	36.5	31.4	17.8	18.7	12.7
CP11	8.1	32.4	138.6	32.0	16.9	f	1	CP	Uni	0	60.1	15.5	3.9	12.2	33.3	29.6	17.0	16.3	13.3
CP12	7.3	32.1	129.3	28.8	19.2	f	1	CP	Uni	1	56.7	14.3	4.0	13.6	31.1	27.3	16.3	14.8	12.5
CP13	11.7	28.5	140.7	30.7	14.4	m	2	CP	Bi	1	43.7	9.6	4.6	12.0	33.0	28.6	14.0	19.0	9.6
TD01	11.7	47.1	156.0	38.2	19.4	f					135.9	22.3	6.1	15.9	41.0	35.3	23.7	17.3	17.9
TD02	11.8	42.9	158.4	36.6	17.1	m					130.8	18.4	7.1	10.8	39.6	32.6	24.9	14.7	18.0
TD03	8.0	28.4	127.7	30.2	17.4	f					64.7	14.0	4.6	13.3	31.7	27.3	19.3	12.4	14.9
TD04	9.7	43.6	153.6	37.6	18.5	f					127.1	21.4	5.9	11.8	39.3	33.5	23.3	16.0	17.5
TD05	10.2	34.8	141.5	32.8	17.4	f					89.3	18.2	4.9	14.0	35.7	30.9	20.0	15.6	15.3
TD06	11.7	41.1	159.0	37.8	16.3	m					107.4	18.0	6.0	12.6	39.3	33.5	20.8	18.5	15.0
TD07	8.6	42.3	154.5	38.7	17.7	m					130.0	24.4	5.3	12.9	39.2	34.0	22.4	16.8	17.2
TD08	10.4	33.0	142.4	32.6	16.3	f					79.5	14.7	5.4	11.2	34.9	29.6	19.9	15.0	14.6
TD09	8.7	29.3	134.7	29.6	16.1	f					91.9	17.4	5.3	14.8	31.5	26.4	19.3	12.2	14.3
TD10	7.2	20.0	120.0	26.3	13.9	m					47.7	11.4	4.2	15.5	28.4	24.5	15.9	12.6	11.9
TD11	9.1	28.1	142.0	32.8	13.9	f					82.5	16.8	4.9	14.3	34.0	29.3	19.4	14.5	14.7
TD12	6.8	23.9	121.8	27.4	16.1	f					55.1	11.9	4.6	13.2	29.4	24.9	17.8	11.6	13.3
TD13	7.5	23.0	123.0	28.3	15.2	f					64.1	13.2	4.8	14.7	29.8	25.2	17.5	12.4	12.8
TD14	8.4	27.2	132.1	30.3	15.6	m					79.9	15.4	5.2	14.1	32.3	27.3	20.1	12.2	15.1

**TD, Typically Developing; CP, Cerebral Palsy; SP-ue, Spastic Paresis of unknown etiology; BoNT-A, Botulinum Neurotoxin A; V_*GM*_*, *muscle volume; A_*fasc*_, physiological cross-sectional area; ℓ_*fasc*_, fascicle length; α_*fasc*_, pennation angle; ℓ_*m*+*t*_, muscle-tendon complex length; ℓ_*a*+*t*_, tendinous structure length; ℓ_*m*_, muscle belly length; ℓ_*t*_, tendon length; ℓ_*a*_, aponeurosis length. ^§^ V_*GM*_ and A_*fasc*_ could not be measured in one SP child.**

### Anthropometry

Body mass and height were measured. Lower leg length was approximated as the mean of distances measured medially and laterally from the most prominent point on each femur epicondyle to the most prominent point of the corresponding malleolus. Patient characteristics are shown individually and summarized in Tables 1, 2, respectively.

**TABLE 2:** Variables of age, subject characteristics and gastrocnemius medialis muscle geometry (at 0 Nm), group comparisons and their correlation with age.

	Group comparison						Coefficient of correlation *r* with age	
		
Variables	TD	SP	df	t	Sig 2-tailed (*p*)	Cohen’s d	TD	SP
Age (years)	9.3 ± 0.5	9.7 ± 0.6	23.4	0.6	0.58	0.22	*N*.*A*	*N*.*A*.
Body mass (kg)	33.2 ± 2.4	33.2 ± 2.7	24.4	0.0	0.99	–0.005	0.83^#^	0.6^#^
Body height (cm)	140.5 ± 3.8	136.8 ± 3.5	49.9	–0.7	0.48	–0.27	0.88^#^	0.72^#^
Lower leg length (cm)	32.8 ± 1.2	31.2 ± 1.1	25.0	–1.0	0.33	–0.38	0.79^#^	0.66^#^
Body mass index (-)	16.5 ± 0.4	17.4 ± 0.7	19.9	1.1	0.29	0.43	0.5	0.34
Sex (# Males/#Females)	5/9	7/6						
# GMFCS per category		I = 3, II = 9, III = 1						
Etiology (CP/SP-ue)		CP = 10, SP-ue = 3						
Distribution (Uni-/Bilateral)		Uni = 2, Bi = 11						
BoNT-A history (naive, 1, > 1)		naive = 4, 1 = 7, > 1 = 2						
V_*GM*_ (ml)^§^	91.8 ± 8.0	52.3 ± 4.1*	19.3	–4.4	< 0.01	–1.65	0.75^#^	0.45
A_*fasc*_ (cm^2^)^§^	17.0 ± 1.0	13.8 ± 1.8	18.0	–1.5	0.14	–0.63	0.59^#^	0.48
ℓ_*fasc*_ (cm)	5.3 ± 0.2	4.1 ± 0.2*	25.0	–4.5	< 0.01	–1.71	0.84^#^	–0.17
α_*fasc*_ (deg)	13.5 ± 0.4	13.9 ± 1.0	16.4	0.4	0.67	0.17	–0.37	0.45
ℓ_*m*+*t*_ (cm)	34.7 ± 1.2	33.0 ± 1.2	24.7	–1.0	0.31	–0.40	0.86^#^	0.69^#^
ℓ_*a*+*t*_ (cm)	29.6 ± 1.0	29.0 ± 1.3	23.0	–0.3	0.74	–0.13	0.82^#^	0.69^#^
ℓ_*m*_ (cm)	20.3 ± 0.7	17.5 ± 0.7*	24.6	–2.8	< 0.01	–1.09	0.77^#^	0.37
ℓ_*t*_ (cm)	14.4 ± 0.6	15.5 ± 0.9	21.6	1.0	0.33	0.39	0.79^#^	0.67^#^
ℓ_*a*_ (cm)	15.2 ± 0.5	13.6 ± 0.8	20.7	–1.7	0.10	–0.68	0.70^#^	0.40

*Indicates a significant difference between groups of Spastic Paresis (SP) and Typically Developing (TD) children (p < 0.05). ^#^Indicates a significant correlation with age (p < 0.05). These symbols indicate the following CP, Cerebral Palsy; SP-ue, Spastic Paresis of unknown etiology; BoNT-A, Botulinum Neurotoxin A; V_*GM*_, muscle volume; A_*fasc*_, physiological cross-sectional area; ℓ_*fasc*_, fascicle length; α_*fasc*_, pennation angle; ℓ_*m*+*t*_, muscle-tendon complex length; ℓ_*a*+*t*_, tendinous structure length; ℓ_*m*_, muscle belly length; ℓ_*t*_, tendon length; ℓ_*a*_, aponeurosis length.***^§^****V_*GM*_ and A_*fasc*_ could not be measured in one SP child.*

### Electromyography

During morphology measurements, surface electromyographies (EMG) of m. gastrocnemius lateralis (GL), because of the inaccessibility of GM, and m. tibialis anterior (TA) were assessed to detect and quantify muscle excitation bursts of agonistic and antagonistic muscles around the ankle. Preparation of the skin and placements of EMG electrodes were performed according to SENIAM guidelines (Hermens et al., 2000), and by determining the outline of the muscles using ultrasound (Hermens et al., 1999). A multichannel system (MOBI, TMS-International, Netherlands) was used to sample EMG signals at 1,024 Hz.

Prior to the measurements, participants were asked to perform a 5-s isometric maximal voluntary contraction (MVC) against resistance (applied by the assessor), toward both dorsal and plantar flexion. During the assessment of ankle moment-angle relation and GM geometry, peaks in the normalized EMG of GL and TA were on average quite low in both TD (GL: 2.6 ± 1.8 %MVC ± SD, TA: 0.8 ± 0.4 %MVC ± SD) and SP (GL: 3.2 ± 1.9 %MVC ± SD, TA: 1.6 ± 1.2 %MVC ± SD) children. Therefore, it is assumed that assessed GM geometry, observed individually, is mainly related to factors other than muscle excitations.

### Foot Plate Angles Corresponding to Externally Exerted Moments

Subjects were asked to lie prone on the examination table with both feet hanging over the edge of the table. Foot plate angles were set by externally applying net moments corresponding to 0 and 4 Nm dorsal flexion. These moments were quantified at the interface between the foot plate and the torque wrench (Bénard et al., 2010). The reproducibility of application of the ankle angle was high (*r* > 0.97). Subsequently, during US scanning the foot plate was fixed to the table at the same angle as during moment application using an extendable rod. Similar to the stabilization procedure of the foot during physical examination, and during the fitting of ankle-foot orthoses, this custom foot plate allows adjustments targeted to stabilize the subtalar joint as much as possible during foot sole rotations (Huijing et al., 2013). In short, the procedure consisted of the following steps: (1) Positioning of the calcaneus in a neutral position under the tibia; (2) Adduction of the forefoot until the midline of the calcaneus points between 2nd and 3rd ray of the forefoot; (3) Applying additional fore and mid-foot supination until no movement within the subtalar joint can be detected by palpation (Huijing et al., 2013).

### GM Morphometry

Comparisons of GM geometry should ideally be made at similar sarcomere lengths. No *in-vivo* approach is available to make such comparisons. GM geometry comparison was made at similar external conditions condition corresponding or relative to a “neutral” foot sole angle. Assuming that fascicles within antagonist muscles are at the same relative length with respect to their optimum length. Ultrasound images were collected at the specific foot plate angles set with the extendable rod corresponding to the standardized externally applied moments (i.e., 0 and 4 Nm, measured before taking the scan). During the 3D ultrasound examination, a 5 cm linear probe (Technos MPX, ESAOTE S.*p*.A. Italy) was moved (swept) longitudinally in a somewhat transverse orientation with respect to the leg over the skin, superficial to the GM. For most subjects multiple sweeps were required to capture the entire GM (Weide et al., 2017). Location and orientation of the probe were registered by a motion capture system (Optotrak 3020, NDI, Waterloo, Canada), and were synchronized with ultrasound images to construct a three-dimensional voxel array, i.e., 3D ultrasound image, using custom software (Matlab, Mathworks Co, Natick, MA, United States) (Weide et al., 2017).

**FIGURE 1:**
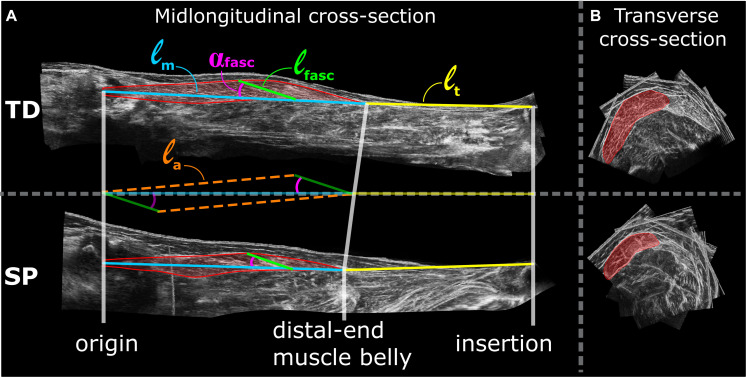
Examples of lower leg 3D ultrasound images from TD (top) and SP (bottom) children. **(A)** left panels show the mid-longitudinal fascicle plane of the gastrocnemius medialis muscle from which fascicle parameters are assessed. Assessments of morphological characteristics are shown as a colored overlay on top of the mid-longitudinal 3D ultrasound image. ℓ_*m*_ = muscle belly length, ℓ_*t*_ = tendon length, α_*fasc*_ = pennation angle, ℓ_*fasc*_ = fascicle length and ℓ_*a*_ = aponeurosis length. **(B)** Right panels show 3D ultrasound images of transverse cross-sections of the GM, hallway along the longitudinal axis of the muscle belly. Red area indicates segmented anatomical cross-section area of the GM from which multiple segmentation along the longitudinal axis of the muscle belly are drawn to estimate muscle volume.

Analysis of 3D ultrasound images was performed using newly adapted methods (Weide et al., 2017), yielding an improvement compared to those used previously by our group (Bénard et al., 2011). Coordinates of the GM insertion on the calcaneus, the most distal end of GM muscle belly, and the estimated coordinates of the GM origin on the medial femur condyle (for anatomical details see Bénard et al., 2010) were assessed from the 3D ultrasound images using the freeware medical imaging interaction toolkit (MITK)^1^. Based on the distances between these coordinates, muscle belly length (ℓ_*m*_), tendon length (ℓ_*t*_), and muscle-tendon complex length (ℓ_*m*+*t*_) were calculated as straight line distances (Figure 1). GM muscle volume (V_*GM*_) was measured between the origin and distal end of the muscle belly using manual segmentation of the anatomical cross-sections and interpolation in MITK. Based on cadaveric experiments (Huijing, 1985), average fascicle length (ℓ_*fasc*_) and pennation angles (α_*fasc*_) were estimated within the mid-longitudinal fascicle plane at a position 2/3rd along the muscle belly (from origin) (Figure 1) which are judged representative for a large fraction of a fascicle of the mid-longitudinal plane. The mid-longitudinal fascicle plane was defined by three points: (1) origin, (2) distal end of the muscle belly, and (3) a point perpendicular to the distal aponeurosis within an anatomical plane at a 2/3rd position along a line segment from point 1 toward point 2 (Weide et al., 2017). Physiological cross-sectional area (A_*fasc*_) was calculated by dividing muscle volume by ℓ_*fasc*_ corresponding to 0 Nm. Aponeurosis length (ℓ_*a*_) was estimated according to the law of cosines of the right triangle constructed by the variables ℓ_*fasc*_, ℓ_*m*_, and α_*fasc*_ (Eq. 1, Figure 1).

(1) ℓa=2ℓf⁢a⁢s⁢c+2ℓm-22*ℓf⁢a⁢s⁢c*ℓm*COS(α)f⁢a⁢s⁢c

Together, the tendon and aponeurosis represent the tendinous structure or the major part of the-series elastic component of GM muscle-tendon complex. We used the summed lengths of these structures (ℓ_*a*+*t*_) as a variable representing the length of the tendinous structure. The extensibility of the total muscle-tendon complex was defined as the absolute change in ℓ_*m*+*t*_ between 0 and 4 Nm conditions.

### Statistical Analysis

Two types of statistics were used:

I. TD-SP group comparisons using means and standard errors. Student’s *t*-tests were used to test for significance of differences between mean values of SP and TD groups regarding age, body mass, body height, lower-leg length and GM geometrical characteristics measured at 0 Nm. Cohen’s D effect size was used to quantitatively represent the difference between the groups. To test for differences in (normalized) morphological characteristics between groups, a two-way mixed ANOVA with between-subject factor (group) and within-subject factors (externally applied moments: 0 and 4 Nm) was used to test for main and interaction effects between groups.
II. Regression analysis was performed using individual data of subjects for each group separately. Pearson’s product-moment coefficients of correlations and linear regressions were used to assess relations between age, and geometrical and anthropometric characteristics, using individual data. Differences in slopes were tested using Sigma Plot (Version 12.0, Systat Software, San Jose, CA).

For Student’s *t*-tests, ANOVA and Pearson’s correlations we used SPSS (version 25.0, SPSS Inc., 2018), with the level of significance set at *p* < 0.05. Strength of relationships were interpreted as weak (*r* = 0–0.3), moderate (*r* = 0.3–0.7) and strong (*r* = 0.7–1).

## Results

### Participant Characteristics

Individual participant characteristics and results are shown in Table 1. There were no differences in age or anthropometric variables between the SP and TD group (Table 2). Body mass increased with increasing lower-leg lengths in both SP and TD (by 2.1 kg cm^–1^ and 2.0 kg cm^–1^ increase in lower-length, respectively).

### Comparisons of GM Geometry of SP and TD Groups Using Mean Data

Since one child with SP moved during the 3DUS imaging procedure, muscle volume could not be measured reliably, and thus V_*GM*_ and A_*fasc*_ could not be determined for this child.

### Absolute GM Geometry (at 0 Nm): SP–TD Comparison

Measured at 0 Nm, muscle volume (V_*GM*_) was on average 47% smaller (−39.6 ml) in children with SP compared to TD children (Table 2). Absolute muscle belly length (ℓ_*m*_) and fascicle length (ℓ_*fasc*_) in the SP group were smaller (by −2.8 cm or −14%, and −1.2 cm or −23%, respectively) compared to the TD group. However, no significant differences could be shown for absolute values of muscle-tendon complex length (ℓ_*m*+*t*_), physiological cross-sectional area (A_*fasc*_), fascicle pennation angle (ℓ_*fasc*_), tendinous structures (ℓ_*a*+*t*_), aponeurosis length (ℓ_*a*_), and tendon length (ℓ_*t*_) measured at 0 Nm (Table 2).

### Normalized GM Geometry (0–4 Nm): SP–TD Comparison

**FIGURE 2:**
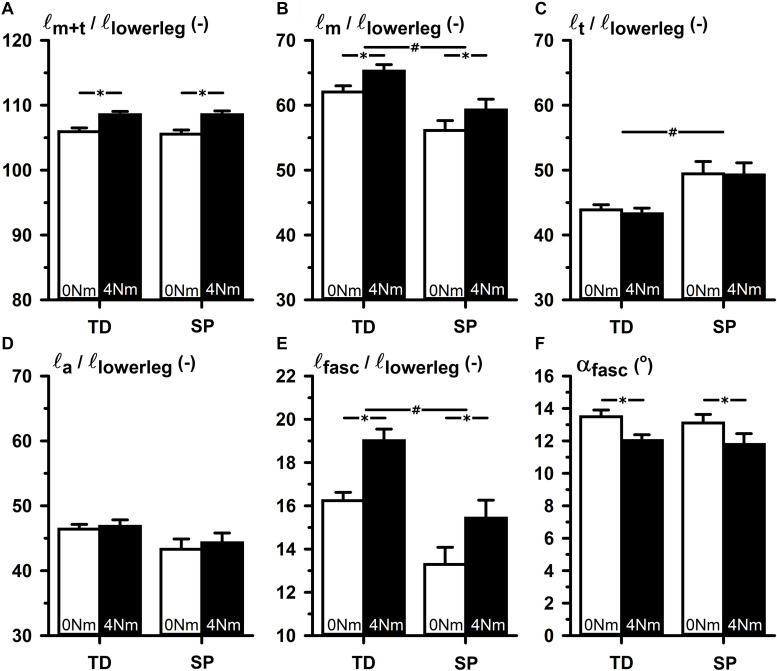
TD-SP comparison of normalized values of elements of GM muscle belly and tendon geometry corresponding to 0 and 4 Nm externally applied dorsal flexion moments. **(A)** ℓ_*m*+*t*_/ℓ_*lowerleg*_: summed lengths of muscle belly and tendon normalized for lower leg length. **(B)** ℓ_*m*_/ℓ_*lowerleg*_: muscle belly length normalized for lower leg length. **(C)** ℓ_*t*_/ℓ_*lowerleg*_: tendon length normalized for lower leg length. **(D)** ℓ_*a*_/ℓ_*lowerleg*_: aponeurosis length normalized for lower leg length. **(E)** ℓ_*fasc*_/ℓ_*lowerleg*_: fascicle length normalized for lower leg length. **(F)** α_*fasc*_: pennation angle in degrees between fascicle and longitudinal axis of the muscle belly. Significance is indicated; *p < 0.05 for a main effect of condition (externally applied dorsal flexion moments), **^#^***p* < 0.05 for a main effect of group (TD-SP).

After normalization for lower leg length, ANOVA of length variables measured at 0 and 4 Nm showed that normalized muscle-tendon complex length (ℓ_*m*+*t*_/ℓ_*lowerleg*_) and normalized aponeurosis length (ℓ_*a*_/ℓ_*lowerleg*_) were not different between groups. However, in SP normalized muscle belly length (ℓ_*m*_/ℓ_*lowerleg*_) was 9.4% shorter and normalized tendon length (ℓ_*t*_/ℓ_*lowerleg*_) 13.3% longer compared to those in the TD group (Figure 2, lines with # indicate the main effects for group differences). In addition, normalized fascicle length (ℓ_*fasc*_/ℓ_*lowerleg*_) was 18.8% shorter in SP compared to TD. Compared to TD, these results showed that after the adjustment for lower leg length differences, muscle-tendon complexes in children with SP seemed to be comprised of a shorter muscle belly, with shorter fascicles, and a longer tendon.

### Effects of Increased Applied Ankle Dorsal Flexion Moments on GM Geometry

In both groups, ℓ_*m*+*t*_/ℓ_*lowerleg*_ increased upon exerting 4 Nm dorsal flexion by 2.8% and ℓ_*m*_/ℓ_*lowerleg*_ increased by 3.2% (Figure 2). In addition, in both groups ℓ_*fasc*_/ℓ_*lowerleg*_ increased by 2.5%, and α_*fasc*_ decreased by 1.4°, similarly. However, in neither of the groups, changes of ℓ_*t*_/ℓ_*lowerleg*_ and ℓ_*a*_/ℓ_*lowerleg*_ were found. There were no interaction effects identified of group and conditions (i.e., between 0 and 4 Nm conditions). These findings showed that changes in GM geometry in response to externally applied ankle dorsal flexion were not different between groups.

### Absolute GM Extensibility (0–4 Nm): SP–TD Comparison

Between groups, no difference was shown for extensibility of GM muscle-tendon complex (Δℓ_*m*+*t*_ = 0.86 cm in TD, Δℓ_*m*+*t*_ = 0.90 cm in SP). However, a somewhat increased variation in extensibility in the SP compared to the TD group (coefficient of variation of 37% in SP and 33% in TD) may have been caused by enhanced heterogeneity within the SP group, but other possibilities should be considered as well.

**FIGURE 3:**
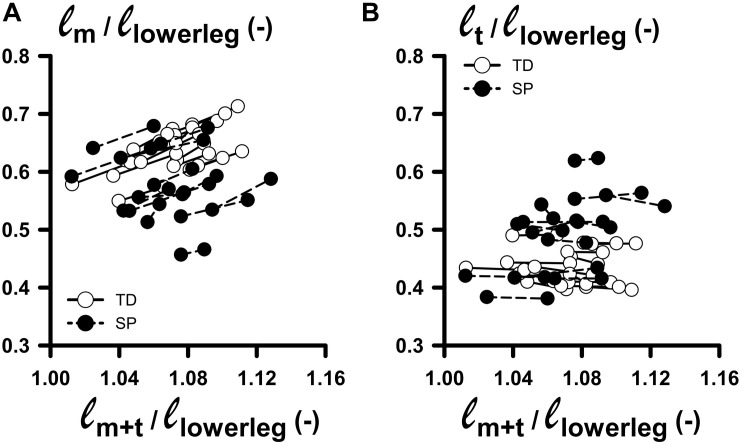
Changes in normalized muscle and tendon lengths as functions of normalized muscle-tendon complex lengths corresponding to externally applied 0–4 Nm dorsal flexion moments. **(A)** Plot relating ℓ_*m*_/ℓ_*lowerleg*:_ (muscle belly length normalized for lower leg length) with ℓ_*m*+*t*_/ℓ_*lowerleg*_ (summed lengths of muscle belly and tendon, normalized for lower leg length) measured at 0 and 4 Nm dorsal flexion moment. **(B)** Plot relating ℓ_*t*_/ℓ_*lowerleg*:_ (tendon length normalized for lower leg length) with ℓ_*m*+*t*_/ℓ_*lowerleg*_, measured at 0 and 4 Nm dorsal flexion moment. Solid line and dashed lines links individual data between 0 and 4 Nm for TD and SP individuals, respectively.

To avoid making group comparisons of length variables corresponding to different conditions, ideally, we should have made comparisons at similar lengths relative to optimum muscle tendon-complex length. However, as measuring optimum length was not feasible, we chose to assess muscle morphology at similar external moments (relative to 0 Nm). We assumed that in this condition the muscle was at the same relative (non-slack) length with respect to optimum length. However, Figure 3 shows large individual variation in normalized muscle and tendon lengths measured at 0 and 4 Nm, yielding overlap for similar lengths conditions only for a limited number of subjects. This, by itself, makes the intended identification of underlying mechanisms of limited extensibility between groups impractical. It also means we should interpret the results of the above group comparisons with the utmost care.

### Comparison of GM Geometrical Variables Using Individual Children’s Data

#### GM Muscle-Tendon Complex Length and Its Constituents (at 0 Nm)

Regression analyses of muscle-tendon complex length (ℓ_*m*+*t*_) with ℓ_*fasc*_, and ℓ_*a*+*t*_ showed that in TD children, ℓ_*fasc*_ significantly and positively correlated with ℓ_*m*+*t*_. However, no such correlation was found for children with SP. In both TD and SP, ℓ_*a*+*t*_ correlated positively with ℓ_*m*+*t*_. These results showed that at 0 Nm for children with SP, longer muscle-tendon complex lengths were accompanied by longer tendons, but not by longer muscle fascicles, as was shown for TD children.

#### GM Muscle Volume and Its Constituents (at 0 Nm)

**FIGURE 4:**
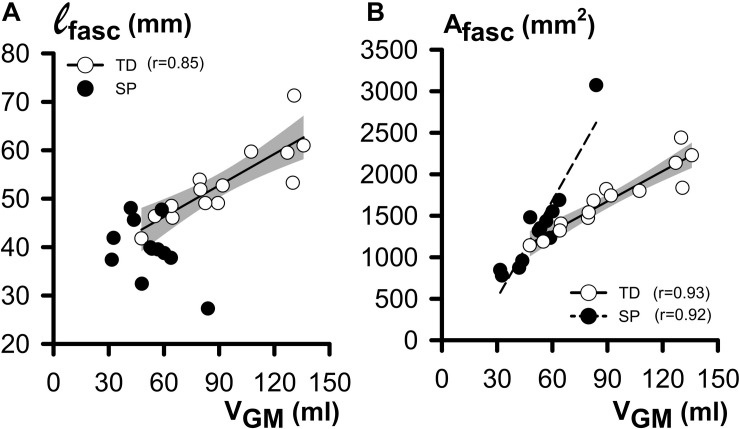
Regression analysis of morphological GM belly characteristics as functions of GM (at 0 Nm). **(A)** Plot relating individual ℓ_*fasc*_ (fascicle length) and V_*GM*_ (muscle volume) data. Note that exclusively for TD children, a significant and positive correlation was shown. **(B)** Plot relating individual A_*fasc*_ (physiological cross-sectional area) and V_*GM*_ data. Note the positive correlation, indicating that in both the TD and SP group, children with a big V_*GM*_ also have a larger A_*fasc*_. The shaded area represents the 95% confidence interval for the TD group. No regression line is drawn and no coefficient of correlation is indicated for data not showing a significant coefficient of correlation.

Regression analyses of GM muscle volume with its constituents ℓ_*fasc*_ and A_*fasc*_ showed that in TD, ℓ_*fasc*_ was significantly and positively correlated with V_*GM*_ (Figure 4A). However, no such correlation was found for children with SP. In both TD and SP, A_*fasc*_ was positively correlated with V_*GM*_ (Figure 4B). One outlying data point may have contributed considerably to these correlations for children with SP, however correlation analysis without this data point was still significant for children with SP (*r* = 0.89).

#### Relations Between Structures Constituting the GM Geometry: SP–TD Comparison

**FIGURE 5:**
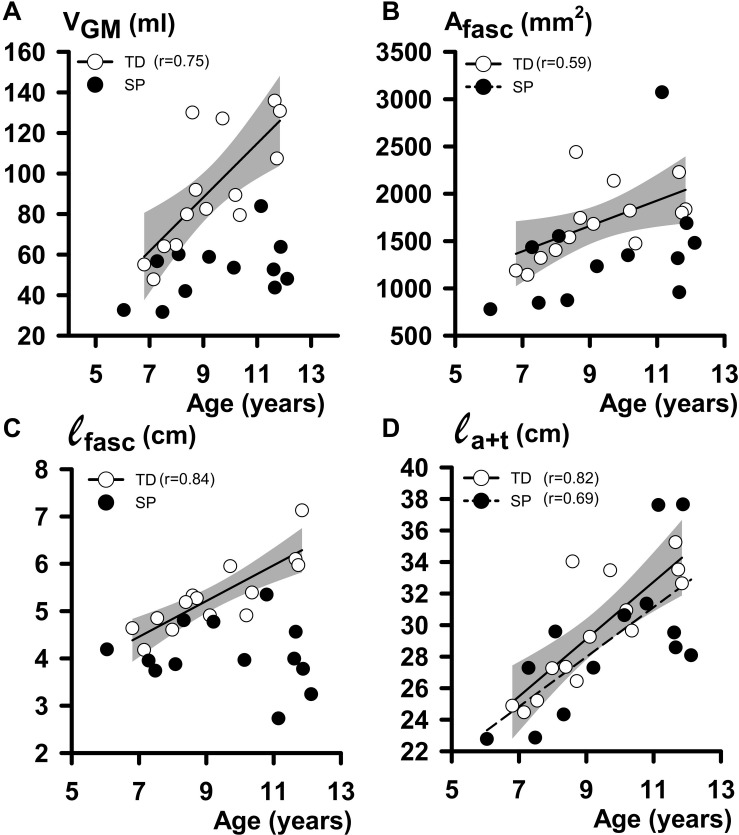
Regression analysis of individual geometrical characteristics of GM muscle and age data. **(A)** Plot relating individual V_*GM*_ (muscle volume at 0 Nm) to age. **(B)** Plot relating individual A_*fasc*_ (physiological cross-sectional area at 0 Nm) to age. **(C)** Plot relating individual ℓ_*fasc*_ (fascicle length at 0 Nm) to age. **(D)** Plot relating individual ℓ_*a*+*t*_ (tendinous structure length at 0 Nm) to age. The shaded area represents the 95% confidence intervals for the TD children. No regression line is drawn, and no coefficient of correlation is indicated for data not showing a significant coefficient of correlation.

In both TD and SP children, age affected GM geometry (Table 2). In TD children, V_*GM*_ increased with age by 13.3 ml year^1^, A_*fasc*_ by 134 mm^2^ year^–1^ and ℓ_*fasc*_ by 3.8 mm year^–1^. However, no significant increases of V_*GM*_, A_*fasc*_, and ℓ_*fasc*_ with age were found for children with SP (Figure 5 and Table 1). Muscle-tendon complex length correlated with age in both TD and SP in a similar way (by 2.2 cm year^–1^ in TD and 1.5 cm year^–1^ and SP, respectively). Tendon length [ℓ_*t*_ increased with age in both TD and SP similarly (by 1.0 cm year^–1^ in TD and 1.0 cm year^–1^ in SP]. Although muscle belly length and aponeurosis length increased in TD with age (increase by 1.2 cm year^–1^, and by 0.78 cm year^–1^, respectively), no such correlations were found in children with SP. Thus, muscle-tendon complex characteristics in TD children were explained by age-related changes of both ℓ_*m*_ and ℓ_*t*_, whereas in children with SP, these characteristics could only be explained by age-related changes in ℓ_*t*_. The length of the tendinous structures (ℓ_*a*+*t*_) increased with age in both TD and SP similarly (by 1.8 cm year^–1^ in TD and 1.6 cm year^–1^ in SP) (Figure 5D).

**FIGURE 6:**
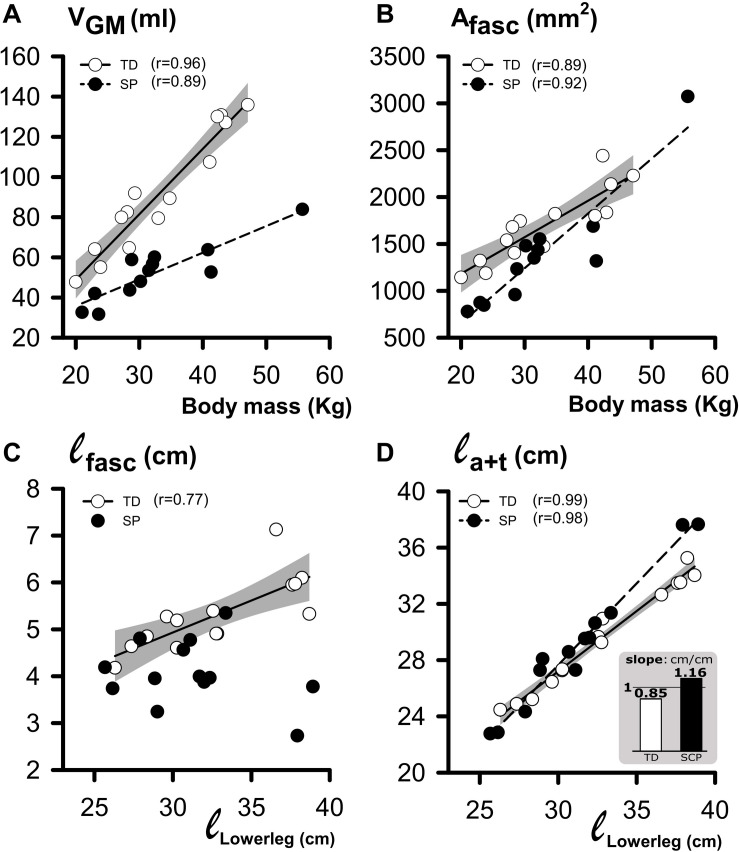
Regression analysis of individual geometrical characteristics of GM muscle and body mass or lower leg length. **(A)** Plot relating individual **V_*GM*_** (muscle volume at 0 Nm) to body mass data. **(B)** Plot relating individual A_*fasc*_ (physiological cross-sectional area at 0 Nm) to body mass data. **(C)** Plot relating individual ℓ_*fasc*_ (fascicle length at 0 Nm) to lower leg length data. **(D)** Plot relating individual ℓ_*a*+*t*_ (i.e., tendinous structure length at 0 Nm) to lower leg length data. The inset bar graph illustrates the significant difference in slope (ℓ_*a*+*t*_/ℓ_*lowerleg*_) between children with SP and TD children. The shaded area represents the 95% confidence interval for the TD group. No regression line is drawn, and no coefficient of correlation is indicated for data not showing a significant coefficient of correlation.

To assess whether GM geometry and its constituents correlated with lower leg length and body mass, we plotted these morphological characteristics as a function of lower leg length and body mass (Figure 6). In TD children and children with SP, muscle volume increased with body mass differently (Figure 6A; increases by 3.3 ml kg^–1^ in TD and 1.3 ml kg^–1^ in SP). In both TD and SP, A_*fasc*_ increased similarly as a function of body mass (by 38.9 mm^2^ kg^–1^ in TD and by 58.7 mm^2^kg^–1^ in SP). For TD children, ℓ_*fasc*_ increased as a function of lower-leg length by 0.14 cm cm^–1^. However, no correlation was found for children with SP (Figure 6C). In TD and SP, ℓ_*a*+*t*_ as a function of lower leg length increased differently (by 0.85 cm cm^–1^ in TD and 1.16 cm cm^–1^ in SP). Thus, length increases of tendinous structures in children with SP exceeded those of TD children. In addition, length increases of the tendinous structures exceeded increases in lower leg length, only in children with SP.

#### Relations of Anthropometric and GM Geometric Variables to GM Extensibility

For both groups, we found no significant correlations between muscle-tendon complex extensibility with age (see Table 3). This indicated that absolute muscle-tendon complex extensibility did not change with age. However, we did find negative correlations between anthropometric variables and muscle-tendon complex extensibility, except for lower leg length. However, no such correlations between anthropometric values and muscle-tendon complex extensibility were found in TD children (Table 3). These findings indicated that only in children with SP increases in body size were associated with decreases in absolute muscle-tendon complex extensibility.

**TABLE 3:** Coefficients of correlation of anthropometric variables and muscle geometrical variables at 0 Nm with muscle-tendon extensibility.

Variables	Coefficient of correlation *r* with extensibility	
	
	TD	SP
Age	0.25	–0.35
Body mass	0.36	−0.61^#^
Body height	0.50	−0.66^#^
Lower leg length	0.51	–0.52
V_*GM*_	0.51	−0.66^#^
A_*fasc*_	0.59^#^	−0.59^#^
ℓ_*fasc*_	0.33	0.44
α_*fasc*_	–0.19	–0.55
ℓ_*m*+*t*_	0.43	–0.54
ℓ_*a*+*t*_	0.43	−0.59^#^
ℓ_*m*_	0.41	–0.10
ℓ_*t*_	0.36	−0.68^#^
ℓ_*a*_	0.42	–0.22

**Significance is indicated;****^#^****p < 0.05. These symbols indicate the following V_*GM*_, muscle volume; A_*fasc*_, physiological cross-sectional area; ℓ_*fasc*_, fascicle length; α_*fasc*_, pennation angle; ℓmt+, muscle-tendon complex length****;***ℓat+, tendinous structure length; ℓ_*m*_, muscle belly length; ℓ_*t*_, tendon length; ℓ_*a*_, aponeurosis length. Muscle-tendon complex extensibility is defined as the absolute change in muscle-tendon complex length from 0 to 4 Nm dorsal flexion.*

Analysis of individual data showed that, in children with SP, a hypothetical 1 mm decrease in GM extensibility was correlated with the net effect of increases in GM muscle volume (by 27.98 ml), A_*fasc*_ (by 1189.38 mm^2^), and length of tendinous structures (by 8.3 cm). In contrast, GM extensibility in TD children was positively correlated to A_*fasc*_. Besides A_*fasc*_, no other GM geometric variable showed a significant correlation with GM extensibility in TD children. These results showed that growth-related increases of GM geometry in children with SP were associated with a decrease in GM extensibility.

## Discussion

Our cross-sectional study shows that the GM geometry of children with SP is mainly characterized by smaller muscle bellies, related to a limited or absent longitudinal fascicle growth. In addition, we found that exclusively in children with SP, growth-related length increases of tendinous structures exceed those of lower leg length. Only in children with SP, we found a negative coefficient of correlation of both the physiological cross-sectional area and length of tendinous structures with muscle-tendon complex extensibility. These results show how at least part of the triceps-surae muscle hyper-resistance to extension in children with SP can be explained by growth-related adaptations of the GM.

### Limitations of the Present Study

Given the design of the present study, we cannot distinguish between mechanisms responsible for acute GM geometry changes or any other structures surrounding the ankle joint in children. It is conceivable that at the exertion of the standardized applied foot plate moments, other structures may be strained, resulting in no changes in GM geometry. Such a mechanism may prevent attainment of higher fascicle lengths or tendon lengths at 0 Nm than those presently found.

Limitations concerning the 3D-ultrasound and inclino-dynamometer technique were described and discussed previously (Bénard et al., 2009, 2010, 2011; Weide et al., 2015, 2017). A major limitation of 3D-ultrasound imaging is that the subjects should not move during the acquisition. Movements result in erroneous displacements of US images in the reconstruction of the 3D voxel array. In addition, during the acquisition, probe pressure results in tissue deformation. To overcome excessive probe pressure, ample ultrasound gel should be applied on the skin. Finally, 3D-ultrasound imaging requires anatomical knowledge to be able to correctly interpret 3D ultrasound image reconstruction. By measuring GM morphology at 0 Nm we assumed that agonistic and antagonistic muscles were not in a slack condition because this would require contraction to pull the series-elastic structure out of the in-toe region, which is very unfavorable.

### Group Comparisons

Studies using ankle-dynamometry measurements and gait analysis show more plantar flexed feet in children with SP compared to TD children, suggesting short muscle-tendon complexes (Tardieu et al., 1982; Harlaar et al., 2000; Singer et al., 2002). In contrast to such studies, we refer to foot sole angles rather than to ankle joint angles, as we have clear indications that foot flexibility confounds the comparison of GM geometry between groups at similar foot sole angles (Huijing et al., 2013; Weide et al., 2020). Ideally, comparisons of GM geometry should be made at comparable sarcomere lengths. Since no *in-vivo* approach is available to make such comparisons, comparisons of GM geometry were standardized to similar external conditions, e.g., “neutral” foot sole angle or externally applied moment. Differences in standardization approach most likely underlie the ambiguity concerning fascicle length in children with SP, since both shorter (Wren et al., 2010) and similar (Schless et al., 2018) muscle-tendon complex lengths have been reported for SP groups. In the present study, there was no difference in muscle-tendon complex length between SP and TD groups. However, it is conceivable that difference in ankle joint range of motion are not exclusively related to GM structures, but also to other structures surrounding the ankle joint. Such interactions between tissues may have prevented the attainment of higher muscle-tendon complex lengths, fascicle lengths, or tendon lengths at 0 Nm than those presently found.

In accordance with other studies that quantified GM geometry at “resting” ankle joint angles or 0 Nm conditions, we did find morphological characteristics such as muscle volume (Barber et al., 2011a; Noble et al., 2014; Pitcher et al., 2018; Schless et al., 2018), fascicle length (Mohagheghi et al., 2008; Gao et al., 2011; Matthiasdottir et al., 2014), and physiological cross-sectional area (Barber et al., 2011a,b) to be smaller, and tendon length (Barber et al., 2012) to be larger in the SP compared to the TD group. However, also in the literature, several studies found similar fascicle lengths at this so-called “resting” joint angle in the SP compared to the TD group (Shortland et al., 2002, 2004; Malaiya et al., 2007; Wren et al., 2010; Barber et al., 2011a,b). Conclusions regarding growth based on group differences may deviate from conclusions based on regression analysis that considers individual variations. This can be exemplified by the study of Malaiya et al. (2007), who studied muscle growth in children with SP and allowed further interpretation due to the transparent and extensive description of their methods and results. Based on group comparison, Malaiya et al. (2007) argued that reduced muscle growth in children with SP is only related to reduced physiological cross-sectional area growth, and not to reduced longitudinal fascicle growth. However, other parts of that same study may lead to different conclusions (see below).

### SP–TD Comparison Based on Individual Data

#### Similar Growth of Body Dimensions in SP and TD Children

Differences in body dimensions between children have been reported (e.g., Grammatikopoulou et al., 2009; Tomoum et al., 2010; Walker et al., 2015; Herskind et al., 2016). In contrast, both body mass and lower leg length and increases thereof over age did not differ between our samples of SP and TD children. This may be due to cross-sectional design and the limited group size. Another explanation may be that the children with SP were relatively mildly affected, as GMFCS levels in SP correlate with smaller body dimensions (Walker et al., 2015; Wang et al., 2016).

#### Less Muscle Volume Growth in Children With SP Compared to TD

In line with the previous studies (Noble et al., 2014; Herskind et al., 2016; Willerslev-Olsen et al., 2018), our results show that GM muscle volume increases with body mass in children with SP, albeit at a reduced rate compared to TD children. It should be noted, however, that at larger body masses, children with SP attain considerably smaller GM muscle volumes than TD children, as the slopes of the regression lines of SP and TD children are very different. In agreement with previous results of Herskind et al. (2016), at similar body mass (~20 kg), GM muscle volumes of children with CP are smaller compared to those of TD children, but the differences seem relatively small. In addition, Herskind et al. (2016) reported that from the age of 15.5 months which is the age at which children typically start to walk, GM muscle volume in children with SP deviates substantially from TD children. This substantial deviation in GM muscle volume is surprising, considering the relatively minor difference in GM muscle volume at ∼20 kg (∼5 years of age). This suggests that, during development, children with SP can catch up with TD children on the aspect of GM muscle volume growth. Further research on how muscles and their mechanical properties develop from birth to adolescence, as well as the mechanisms underlying the attenuated muscle growth in young and older children with SP is indicated.

#### Increases of GM Tendinous Structures in SP and TD Children

Particularly at older ages, or larger lower leg lengths, the tendinous structure in children with SP is relatively longer than in TD children. This is emphasized by the observation that exclusively in children with SP increases in tendinous structure lengths exceeded increases in lower leg length. A similar effect, namely increases in tendinous lengths being larger than bone growth, was also seen following an experimental transfer of the flexor m. carpi-ulnaris distal tendon to the extensor site in healthy animals (Maas and Huijing, 2012). Thus, our present results confirm indications of other studies (Tardieu et al., 1977; Wren et al., 2010), that in children with SP, factors other than lower leg length must be involved in regulating tendon growth.

Mechanisms responsible for the adaptation of tendinous structures potentially change with maturation. In young animals, it has been shown that the muscle-tendon complex adapts its length to immobilization by tendon length changes, without changes in the number of sarcomeres in series (Tardieu et al., 1977; Blanchard et al., 1985). However, in adult animals, the muscle-tendon complex adapts solely by changes in the number of sarcomeres in series (Tardieu et al., 1977; Blanchard et al., 1985; Wren, 2003). It has been suggested that at a younger age, lengths of tendons adapt to minimize strain (Wren, 2003). Increasing the length of tendinous structures to reduce strain in children with SP may be beneficial to allow more ankle joint movement in the short term. However, the capacity of tendon structures to adapt in such a way seems to diminish in adult age for unknown reasons (Blanchard et al., 1985). One explanation may be a decreasing expression of growth factors responsible for tendon growth in young children (Okamoto et al., 2005; Gumucio et al., 2015). Especially in children with SP, higher concentrations of transforming growth factor beta (TGF-β1) have been shown within the muscle and in the serum compared to TD children and adults (Grether et al., 1999; Lin et al., 2010; Von Walden et al., 2018; Pingel et al., 2019). Such age dependent tendon plasticity and elevated expression of TGF-β1 in SP compared to TD children may result in altered morphological and mechanical tendon properties in young children with SP.

If the material properties of the tendinous structures and the cross-sectional area remain similar, it is expected that the overall structure compliance increases with an increase in slack length. However, our results show that tendon length in children with SP correlates negatively with muscle-tendon complex extensibility. This finding indicates that during growth, tendon material properties change, and/or the length of tendinous structures increases. When stretching the muscle in both SP and TD children by applying an externally applied moment (4 Nm), we did not find [acute] increases in the summed length of tendinous structures, nor for tendon or aponeurosis separately. Even when exerting 0 Nm externally, non-zero stresses on the Achilles tendon, its aponeuroses, and on tendinous structures of antagonistic muscles are expected (Wu et al., 2012), so that at 0 and 4 Nm these structures are on the stiff part of their length-force curves. Fast release experiments on maximally dissected animal muscles have shown that tendinous structures (rather than intra-fiber components) lengthen only by 4% from zero force to optimum muscle force (Morgan et al., 1978). Some simple and rough calculations may guide our expectations on this (see next paragraph).

#### Analysis of Effects on Tendinous Extensibility in TD Children

In a TD child with a tendinous structure length (ℓ_*a*+*t*_) of ~30 cm and an optimum active force of 510 *N* (17 cm^2^ of A_*fasc*_ and a specific tension of around 30 *N*/cm^2^; Erskine et al., 2011; van der Zwaard et al., 2018), we would expect to find 1.2 cm (4% of 30 cm) of tendon stretch. If dorsal flexion resistance to an external moment of 4 Nm should originate solely from GM, with an estimated moment arm ~5 cm (Kalkman et al., 2017), it would cause a force pulling on the tendon with 80 *N* (F = ^4^/_0.05_ *N*). Assuming a linear stress-strain curve, 80 *N* of force pulling on the tendon would result in 0.2 cm (^80^/_510_^∗^1.2) stretch of the tendinous structure. However, in reality, other parallel arranged structures provide additional force transmission pathways, such as other plantar flexor muscles, joint capsules and ligaments, reducing the above estimated stress and strain of the GM tendinous structures. Thus, our finding of negligible acute increases length of tendinous structures in response to the small and low range of externally applied moment meets our expectations. Unfortunately, we could not capture these 2 mm acute increases in tendon length because of the limited sensitivity of the 3D US setup.

#### Lower Fascicle Lengths Attained in SP Compared to TD Children

In our sample of children with SP, age range 6–13 years, fascicle length expressed as functions of either age, lower leg length or muscle volume, deviates from those in TD children. However, differences in fascicle length between children with SP and TD children were small at a lower age, shorter lower legs, and smaller muscle volumes. This is in agreement with the results of Herskind et al. (2016), who reported fascicle length of SP and TD children up to five years old to increase indifferently. However, after this age, differences in fascicle length between SP and TD increase with increases in age and lower leg length. Such a deviation is also reported in the study by Malaiya et al. (2007) showing that in TD children, but not in children with SP, individual fascicle length at “resting” foot angle correlates with fibula length. However, the finding that fascicle length only increases in TD seems underappreciated in their conclusion regarding GM muscle growth in children with SP, stating that reduced GM growth is related to a reduced physiological cross-sectional area growth.

Reduced longitudinal fascicle growth in children with SP may be related to reduced addition of sarcomeres in series, resulting in a smaller number of sarcomeres in series (Mathewson et al., 2015) and/or longer sarcomeres (Smith et al., 2011; Mathewson et al., 2015). Several mechanisms may be responsible for the comparatively reduced addition of sarcomeres in series in children with SP (Dayanidhi and Lieber, 2014; Von Walden et al., 2018). Possibly in children with SP, this may be due to the effects of spasticity hindering typical muscle use and proliferation and differentiation of satellite cells (Dayanidhi and Lieber, 2014). Alternatively, enhanced longitudinal tendon growth in children with SP may attenuate the stimulus for longitudinal fascicle growth, resulting in a reduced number of sarcomeres arranged in series. A reduced number of sarcomeres arranged in series negatively corresponds to the extensibility of the muscle fascicle.

#### Physiological Cross-Sectional Area Growth in SP and TD Children

In children with SP and TD, the physiological cross-sectional area of GM increases similarly with body mass. Such conclusions are also supported by Malaiya et al. (2007) after the reinterpretation of their data showing that in children with SP, GM muscle volumes increase without increases in fascicle length. Therefore, it can be concluded that in children with SP, at least in the age range of 6–13 years, increases in muscle volume are mainly caused by increases in physiological cross-sectional area.

One may predict that increases in GM physiological cross-sectional area would negatively affect extensibility of the muscle-tendon complex, as more parallel arranged muscular material needs to be strained and may affect ankle range of motion (Weide et al., 2015). Understanding how different underlying mechanisms contribute to the measured net extensibility is complex. For example, in our TD children, we found a positive coefficient of correlation between the physiological cross-sectional area and the muscle-tendon complex extensibility. In TD children, the negative effects of the increase of the physiological cross-sectional area on the extensibility may be compensated by simultaneous increases of fascicle length, which is not the case in children with SP.

## Conclusion

In conclusion, this cross-sectional study indicates that age-related GM growth in children with SP is characterized by increases in physiological cross-sectional area and in lengths of tendinous structures, without an increase in fascicle length. Our results show that in children with SP, increases in GM muscle volume, physiological cross-sectional and lengths of tendinous structures are associated with a reduced GM extensibility, while such relations were not shown in TD. The findings of this study suggest that clinical interventions treating hyper ankle dorsiflexion resistance should aim to increase both muscle physiological cross-sectional area and fascicle lengths. Longitudinal 3D ultrasound studies of the morphology and extensibility of the TS muscle are required to verify the present results and to obtain insight in the mechanisms underlying the impeded growth in children with SP.

## Data Availability Statement

The datasets generated for this study are available on request to the corresponding author.

## Ethics Statement

The studies involving human participants were reviewed and approved by the Ethics Committee of the Amsterdam UMC (location VUmc, Amsterdam, Netherlands). Written informed consent to participate in this study was provided by the participants’ legal guardian/next of kin.

## Author Contributions

GW, PH, LB-O, LS, AB, JB, JH, and RJ conceived and designed the work, analyzed and interpreted the data, and drafted and revised the manuscript. GW, LB-O, and LS acquired the data. All authors contributed to the article and approved the submitted version.

## Conflict of Interest

The authors declare that the research was conducted in the absence of any commercial or financial relationships that could be construed as a potential conflict of interest.

## Footnotes

1 www.mitk.org

## References

[B1] Ballaz L. Plamondon S. Lemay M. (2010). Ankle range of motion is key to gait efficiency in adolescents with cerebral palsy. *Clin. Biomech.* 25 944–948. 10.1016/j.clinbiomech.2010.06.011 20655641

[B2] Barber L. Barrett R. Lichtwark G. (2011a). Passive muscle mechanical properties of the medial gastrocnemius in young adults with spastic cerebral palsy. *J. Biomech.* 44 2496–2500. 10.1016/j.jbiomech.2011.06.008 21762920

[B3] Barber L. Barrett R. Lichtwark G. (2012). Medial gastrocnemius muscle fascicle active torque-length and Achilles tendon properties in young adults with spastic cerebral palsy. *J. Biomech.* 45 2526–2530. 10.1016/j.jbiomech.2012.07.018 22867763

[B4] Barber L. Hastings-Ison T. Baker R. Barrett R. Lichtwark G. (2011b). Medial gastrocnemius muscle volume and fascicle length in children aged 2 to 5years with cerebral palsy. *Dev. Med. Child Neurol.* 53 543–548. 10.1111/j.1469-8749.2011.03913.x 21506995

[B5] Barber L. A. Read F. Lovatt Stern J. Lichtwark G. Boyd R. N. (2016). Medial gastrocnemius muscle volume in ambulant children with unilateral and bilateral cerebral palsy aged 2 to 9 years. *Dev. Med. Child Neurol.* 58 1146–1152. 10.1111/dmcn.13132 27098082

[B6] Barrett R. S. Lichtwark G. A. (2010). Gross muscle morphology and structure in spastic cerebral palsy: a systematic review. *Dev. Med. Child Neurol.* 52 794–804. 10.1111/j.1469-8749.2010.03686.x 20477832

[B7] Beckung E. Carlsson G. Carlsdotter S. Uvebrant P. (2007). The natural history of gross motor development in children with cerebral palsy aged 1 to 15 years. *Dev. Med. Child Neurol.* 49 751–756. 10.1111/j.1469-8749.2007.00751.x 17880644

[B8] Bénard M. R. Becher J. G. Harlaar J. Huijing P. A. Jaspers R. T. (2009). Anatomical information is needed in ultrasound imaging of muscle to avoid potentially substantial errors in measurement of muscle geometry. *Muscle Nerve* 39 652–665. 10.1002/mus.21287 19291798

[B9] Bénard M. R. Harlaar J. Becher J. G. Huijing P. A. Jaspers R. T. (2011). Effects of growth on geometry of gastrocnemius muscle in children: a three-dimensional ultrasound analysis. *J. Anat.* 219 388–402. 10.1111/j.1469-7580.2011.01402.x 21635250 PMC3171775

[B10] Bénard M. R. Jaspers R. T. Huijing P. A. Becher J. G. Harlaar J. (2010). Reproducibility of hand-held ankle dynamometry to measure altered ankle moment-angle characteristics in children with spastic cerebral palsy. *Clin. Biomech.* 25 802–808. 10.1016/j.clinbiomech.2010.04.010 20541856

[B11] Blanchard O. Cohen-Solal L. Tardieu C. Allain J. C. Tabary C. Lous M. L. (1985). Tendon adaptation to different long term stresses and collagen reticulation in soleus muscle. *Connect. Tissue Res.* 13 261–267. 10.3109/03008208509152405 3159543

[B12] Dallmeijer A. J. Baker R. Dodd K. J. Taylor N. F. (2011). Association between isometric muscle strength and gait joint kinetics in adolescents and young adults with cerebral palsy. *Gait Post.* 33 326–332. 10.1016/j.gaitpost.2010.10.092 21185726

[B13] Dayanidhi S. Lieber R. L. (2014). Skeletal muscle satellite cells: mediators of muscle growth during development and implications for developmental disorders. *Muscle Nerve* 50 723–732. 10.1002/mus.24441 25186345 PMC4206584

[B14] Erskine R. M. Jones D. A. Maffulli N. Williams A. G. Stewart C. E. Degens H. (2011). What causes in vivo muscle specific tension to increase following resistance training? *Exp. Physiol.* 96 145–155. 10.1113/expphysiol.2010.053975 20889606

[B15] Fry N. R. Gough M. McNee A. E. Shortland A. P. (2007). Changes in the volume and length of the medial gastrocnemius after surgical recession in children with spastic diplegic cerebral palsy. *J. Pediatr. Orthop.* 27 769–774. 10.1097/BPO.0b013e3181558943 17878783

[B16] Gage J. R. (2009). *The Identification and Treatment of Gait Problems in Cerebral Palsy*, 2nd Edn Cambridge: Mac Keith Press.

[B17] Gao F. Zhao H. Gaebler-Spira D. Zhang L. Q. (2011). In vivo evaluations of morphologic changes of gastrocnemius muscle fascicles and Achilles tendon in children with cerebral palsy. *Am. J. Phys. Med. Rehabil.* 90 364–371. 10.1097/PHM.0b013e318214f699 21765255

[B18] Grammatikopoulou M. G. Daskalou E. Tsigga M. (2009). Diet, feeding practices, and anthropometry of children and adolescents with cerebral palsy and their siblings. *Nutrition* 25 620–626. 10.1016/j.nut.2008.11.025 19216055

[B19] Grether J. K. Nelson K. B. Dambrosia J. M. Phillips T. M. (1999). Interferons and cerebral palsy. *J. Pediatr.* 134 324–332. 10.1016/S0022-3476(99)70458-0 10064670

[B20] Gumucio J. P. Sugg K. B. Mendias C. L. (2015). TGF-β superfamily signaling in muscle and tendon adaptation to resistance exercise. *Exerc. Sport Sci. Rev.* 43 93–99. 10.1249/JES.0000000000000041 25607281 PMC4369187

[B21] Harlaar J. Becher J. G. Snijders C. J. Lankhorst G. J. (2000). Passive stiffness characteristics of ankle plantar flexors in hemiplegia. *Clin. Biomech.* 15 261–270. 10.1016/S0268-0033(99)00069-8 10675667

[B22] Hermens H. J. Disselhorst-Klug C. Rau G. (1999). “The recommendations for sensors and sensor placement procedures for surface electromyography,” in *SENIAM 8; European Recommendations for Surface Electromyography*, eds Hermens H. Freriks B. Merletti R. (Roessinghsbleekweg: Roessingh Research and Development), 13–54.

[B23] Hermens H. J. Freriks B. Disselhorst-Klug C. Rau G. (2000). Development of recommendations for SEMG sensors and sensor placement procedures. *J. Electromyogr. Kinesiol* 10 361–374. 10.1016/S1050-6411(00)00027-4 11018445

[B24] Herskind A. Ritterband-Rosenbaum A. Willerslev-Olsen M. Lorentzen J. Hanson L. Lichtwark G. (2016). Muscle growth is reduced in 15-month-old children with cerebral palsy. *Dev. Med. Child Neurol.* 58 485–491. 10.1111/dmcn.12950 26510820

[B25] Huijing P. A. (1985). Architecture of the Human Gastrocnemius Muscle and Some Functional Consequences. *Cells Tissues Organs* 123 101–107. 10.1159/000146047 4061024

[B26] Huijing P. A. Bénard M. R. Harlaar J. Jaspers R. T. Becher J. G. (2013). Movement within foot and ankle joint in children with spastic cerebral palsy: a 3-dimensional ultrasound analysis of medial gastrocnemius length with correction for effects of foot deformation. *BMC Musculoskelet. Disord.* 14:365. 10.1186/1471-2474-14-365 24364826 PMC3909357

[B27] Kalkman B. M. Bar-On L. Cenni F. Maganaris C. N. Bass A. Holmes G. (2017). Achilles tendon moment arm length is smaller in children with cerebral palsy than in typically developing children. *J. Biomech.* 56 48–54. 10.1016/j.jbiomech.2017.02.027 28318605

[B28] Koman L. A. Smith B. P. Shilt J. S. (2004). Cerebral palsy. *Lancet* 363 1619–1631. 10.1016/S0140-6736(04)16207-7 15145637

[B29] Lieber R. L. Runesson E. Einarsson F. Fridén J. (2003). Inferior mechanical properties of spastic muscle bundles due to hypertrophic but compromised extracellular matrix material. *Muscle Nerve* 28 464–471. 10.1002/mus.10446 14506719

[B30] Lin C. Y. Chang Y. C. Wang S. T. Lee T. Y. Lin C. F. Huang C. C. (2010). Altered inflammatory responses in preterm children with cerebral palsy. *Ann. Neurol.* 68 204–212. 10.1002/ana.22049 20695013

[B31] Maas H. Huijing P. A. (2012). Effects of tendon and muscle belly dissection on muscular force transmission following tendon transfer in the rat. *J. Biomech.* 45 289–296. 10.1016/j.jbiomech.2011.10.026 22093795

[B32] Malaiya R. McNee A. E. Fry N. R. Eve L. C. Gough M. Shortland A. P. (2007). The morphology of the medial gastrocnemius in typically developing children and children with spastic hemiplegic cerebral palsy. *J. Electromyogr. Kinesiol.* 17 657–663. 10.1016/j.toxicon.2014.07.016 17459729

[B33] Marsden J. Ramdharry G. Stevenson V. Thompson A. (2012). Muscle paresis and passive stiffness: key determinants in limiting function in hereditary and sporadic spastic paraparesis. *Gait Post.* 35 266–271. 10.1016/j.gaitpost.2011.09.018 22050971 PMC3657152

[B34] Mathewson M. A. Lieber R. L. (2015). Pathophysiology of muscle contractures in cerebral palsy. *Phys. Med. Rehabil. Clin. North Am.* 26 57–67. 10.1016/j.pmr.2014.09.005 25479779 PMC4258234

[B35] Mathewson M. A. Ward S. R. Chambers H. G. Lieber R. L. (2015). High resolution muscle measurements provide insights into equinus contractures in patients with cerebral palsy. *J. Orthop. Res.?* 33 33–39. 10.1002/jor.22728 25242618 PMC4343320

[B36] Matthiasdottir S. Hahn M. Yaraskavitch M. Herzog W. (2014). Muscle and fascicle excursion in children with cerebral palsy. *Clin. Biomech.* 29 458–462. 10.1016/j.clinbiomech.2014.01.002 24485882

[B37] Mohagheghi A. A. Khan T. Meadows T. H. Giannikas K. Baltzopoulos V. Maganaris C. N. (2008). In vivo gastrocnemius muscle fascicle length in children with and without diplegic cerebral palsy. *Dev. Med. Child Neurol.* 50 44–50. 10.1111/j.1469-8749.2007.02008.x 18173630

[B38] Morgan D. L. Proske U. Warren D. (1978). Measurements of muscle stiffness and the mechanism of elastic storage of energy in hopping kangaroos. *J. Physiol.* 282 253–261. 10.1113/jphysiol.1978.sp012461 722527 PMC1282737

[B39] Nieuwenhuys A. Papageorgiou E. Pataky T. De Laet T. Molenaers G. Desloovere K. (2016). Literature review and comparison of two statistical methods to evaluate the effect of botulinum toxin treatment on gait in children with cerebral palsy. *PLoS One* 11:e0152697. 10.1371/journal.pone.0152697 27030973 PMC4816309

[B40] Noble J. J. Fry N. R. Lewis A. P. Keevil S. F. Gough M. Shortland A. P. (2014). Lower limb muscle volumes in bilateral spastic cerebral palsy. *Brain Dev.* 36 294–300. 10.1016/J.BRAINDEV.2013.05.008 23790825

[B41] Nordmark E. Hägglund G. Lauge-Pedersen H. Wagner P. Westbom L. (2009). Development of lower limb range of motion from early childhood to adolescence in cerebral palsy: a population-based study. *BMC Med.* 7:65. 10.1186/1741-7015-7-65 19863779 PMC2774339

[B42] Okamoto Y. Gotoh Y. Uemura O. Tanaka S. Ando T. Nishida M. (2005). Age-dependent decrease in serum transforming growth factor (TGF)-beta 1 in healthy Japanese individuals. Population study of serum TGF-beta 1 level in Japanese. *Dis. Mark.* 21 71–74. 10.1155/2005/381215 15920293 PMC3850592

[B43] Parodi L. Coarelli G. Stevanin G. Brice A. Durr A. (2018). Hereditary ataxias and paraparesias: clinical and genetic update. *Curr. Opin. Neurol.* 31 462–471. 10.1097/WCO.0000000000000585 29847346

[B44] Pingel J. Barber L. Andersen I. T. Von Walden F. Wong C. Døssing S. (2019). Systemic inflammatory markers in individuals with cerebral palsy. *Eur. J. Inflamm.* 17:205873921882347 10.1177/2058739218823474

[B45] Pitcher C. A. Elliott C. M. Valentine J. P. Stannage K. Williams S. A. Shipman P. J. (2018). Muscle morphology of the lower leg in ambulant children with spastic cerebral palsy. *Muscle Nerve* 58 818–823. 10.1002/mus.26293 29981242

[B46] Rosenbaum P. Paneth N. Leviton A. Goldstein M. Bax M. Damiano D. (2007). A report‘he definition and classification of cerebral palsy April 2006. *Dev. Med. Child Neurol.* 109 8–14. 10.1111/j.1469-8749.2007.tb12610.x 17370477

[B47] Schless S. H. Hanssen B. Cenni F. Bar-On L. Aertbeliën E. Molenaers G. (2018). Estimating medial gastrocnemius muscle volume in children with spastic cerebral palsy: a cross-sectional investigation. *Dev. Med. Child Neurol.* 60 81–87. 10.1111/dmcn.13597 29067675

[B48] Shortland A. P. Harris C. A. Gough M. Robinson R. O. (2002). Architecture of the medial gastrocnemius in children with spastic diplegia. *Dev. Med. Child Neurol.* 44 796–801. 10.1017/s0012162201001864 11769264

[B49] Shortland A. P. M. Fry N. Eve L. Gough M. (2004). Changes to medial gastrocnemius architecture after surgical intervention in spastic diplegia. *Dev. Med. Child Neurol.* 46 667–673. 10.1017/S0012162204001124 15473170

[B50] Singer B. Dunne J. Singer K. P. Allison G. (2002). Evaluation of triceps surae muscle length and resistance to passive lengthening in patients with acquired brain injury. *Clin. Biomech.* 17 152–161. 10.1016/S0268-0033(01)00116-4 11832266

[B51] Smith L. R. Lee K. S. Ward S. R. Chambers H. G. Lieber R. L. (2011). Hamstring contractures in children with spastic cerebral palsy result from a stiffer extracellular matrix and increased in vivo sarcomere length. *J. Physiol.* 589(Pt. 10), 2625–2639. 10.1113/jphysiol.2010.203364 21486759 PMC3115830

[B52] Spijker M. Strijers R. L. M. van Ouwerkerk W. J. R. Becher J. G. (2009). Disappearance of spasticity after selective dorsal rhizotomy does not prevent muscle shortening in children with cerebral palsy: a case report. *J. Child Neurol.* 24 625–627. 10.1177/0883073808325652 19151363

[B53] Tardieu C. Huet de la Tour E. Bret M. D. Tardieu G. (1982). Muscle hypoextensibility in children with cerebral palsy: I. *Clinical and experimental observations*. *Arch. Phys. Med. Rehabil.* 63 97–102. 7073456

[B54] Tardieu C. Tabary J. C. Tabary C. Huet de la Tour E. (1977). Comparison of the sarcomere number adaptation in young and adult animals. Influence of tendon adaptation. *J. Physiol.* 73 1045–1055. 615249

[B55] Tedroff K. Granath F. Forssberg H. Haglund-Akerlind Y. (2009). Long-term effects of botulinum toxin A in children with cerebral palsy. *Dev. Med. Child Neurol.* 51 120–127. 10.1111/j.1469-8749.2008.03189.x 19191845

[B56] Tomoum H. Y. Badawy N. B. Hassan N. E. Alian K. M. (2010). Anthropometry and body composition analysis in children with cerebral palsy. *Clin. Nutr.* 29 477–481. 10.1016/j.clnu.2009.10.009 19926178

[B57] van den Noort J. C. Bar-On L. Aertbeliën E. Bonikowski M. Braendvik S. M. Broström E. W. (2017). European consensus on the concepts and measurement of the pathophysiological neuromuscular responses to passive muscle stretch. *Eur. J. Neurol.* 24 981–e38. 10.1111/ene.13322 28557247

[B58] van der Zwaard S. van der Laarse W. J. Weide G. Bloemers F. W. Hofmijster M. J. Levels K. (2018). Critical determinants of combined sprint and endurance performance: an integrative analysis from muscle fiber to the human body. *FASEB J.* 32 2110–2123. 10.1096/fj.201700827R 29217665

[B59] Von Walden F. Gantelius S. Liu C. Borgström H. Björk L. Gremark O. (2018). Muscle contractures in patients with cerebral palsy and acquired brain injury are associated with extracellular matrix expansion, pro-inflammatory gene expression, and reduced rRNA synthesis. *Muscle Nerve* 58 277–285. 10.1002/mus.26130 29572878

[B60] Voorman J. M. Dallmeijer A. J. Knol D. L. Lankhorst G. J. Becher J. G. (2007). Prospective longitudinal study of gross motor function in children with cerebral palsy. *Arch. Phys. Med. Rehabil.* 88 871–876. 10.1016/j.apmr.2007.04.002 17601467

[B61] Walker J. L. Bell K. L. Stevenson R. D. Weir K. A. Boyd R. N. Davies P. S. W. (2015). Differences in body composition according to functional ability in preschool-aged children with cerebral palsy. *Clin. Nutr.* 34 140–145. 10.1016/j.clnu.2014.02.007 24613145

[B62] Wang F. Cai Q. Shi W. Jiang H. Li N. Ma D. (2016). A cross-sectional survey of growth and nutritional status in children with cerebral palsy in west China. *Pediatr. Neurol.* 58 90–97. 10.1016/j.pediatrneurol.2016.01.002 27268760

[B63] Weide G. Huijing P. A. Becher J. G. Jaspers R. T. Harlaar J. (2020). Foot flexibility confounds the assessment of triceps surae extensibility in children with spastic paresis during typical physical examinations. *J. Biomech.* 99:109532. 10.1016/j.jbiomech.2019.109532 31879075

[B64] Weide G. Huijing P. A. P. A. Maas J. C. J. C. Becher J. G. J. G. Harlaar J. Jaspers R. T. R. T. (2015). Medial gastrocnemius muscle growth during adolescence is mediated by increased fascicle diameter rather than by longitudinal fascicle growth. *J. Anat.* 226 530–541. 10.1111/joa.12306 25879671 PMC4450957

[B65] Weide G. van der Zwaard S. Huijing P. A. Jaspers R. T. Harlaar J. (2017). 3D ultrasound imaging: fast and cost-effective morphometry of musculoskeletal tissue. *J. Vis. Exp.* 2017:e55943. 10.3791/55943 29286445 PMC5755508

[B66] Willerslev-Olsen M. Choe Lund M. Lorentzen J. Barber L. Kofoed-Hansen M. Nielsen J. B. (2018). Impaired muscle growth precedes development of increased stiffness of the triceps surae musculotendinous unit in children with cerebral palsy. *Dev. Med. Child Neurol.* 60 672–679. 10.1111/dmcn.13729 29573407

[B67] Woittiez R. Huijing P. Rozendal R. (1983). Influence of muscle architecture on the length-force diagram of mammalian muscle. *Pflueg. Arch.* 399 275–279. 10.1007/bf00652752 6664830

[B68] Wren T. A. L. (2003). A computational model for the adaptation of muscle and tendon length to average muscle length and minimum tendon strain. *J. Biomech.* 36 1117–1124. 10.1016/S0021-9290(03)00107-6 12831737

[B69] Wren T. A. L. Cheatwood A. P. Rethlefsen S. A. Hara R. Perez F. J. Kay R. M. (2010). Achilles tendon length and medial gastrocnemius architecture in children with cerebral palsy: and equinus gait. *J. Pediatric Orthop.* 30 479–484. 10.1097/BPO.0b013e3181e00c80 20574267

[B70] Wu M. Pai D. K. Tresch M. C. Sandercock T. G. (2012). Passive elastic properties of the rat ankle. *J. Biomech.* 45 1728–1732. 10.1016/j.jbiomech.2012.03.017 22520588 PMC3725263

[B71] Zuurbier C. J. Huijing P. A. (1993). Changes in geometry of actively shortening unipennate rat gastrocnemius muscle. *J. Morphol.* 218 167–180. 10.1002/jmor.1052180206 8263946

